# Characteristics and outcomes of ECMO cannula-related infections: a European multicenter retrospective study

**DOI:** 10.1186/s13613-025-01446-y

**Published:** 2025-03-20

**Authors:** Sofia Ortuno, Nicolas Massart, Charles Vidal, Etienne de Montmollin, Adrien Bouglé, Nicolas Nesseler, Frank Bidar, Benjamin Assouline, Paul Masi, Samuel Henri, Sami Hraiech, Hadrien Rozé, Francesca Manicone, Charles-Edouard Luyt

**Affiliations:** 1 Service de Médecine Intensive–Réanimation, Institut de Cardiologie, Groupe Hospitalier Pitié-Salpêtrière, Sorbonne Université, Assistance Publique-Hôpitaux de Paris, 47-83, Boulevard de L’Hôpital, 75651 Paris Cedex 13, France; 2 Service de Réanimation, CH de Saint-Brieuc, Saint-Brieuc, France; 3 Réanimation Polyvalente, Centre Hospitalier Universitaire de La Réunion Site Félix Guyon, Saint-Denis, France; 4 Service de Médecine Intensive–Réanimation, Hôpital Bichat-Claude Bernard, Assistance Publique-Hôpitaux de Paris, Paris, France; 5 Département d’Anesthésie – Réanimation, GRC 29, Institut de Cardiologie, Groupe Hospitalier Pitié-Salpêtrière, Sorbonne Université, Assistance Publique-Hôpitaux de Paris, Paris, France; 6 Department of Anesthesia and Critical Care, Pontchaillou, University Hospital of Rennes, Rennes, France; 7 Univ Rennes, CHU Rennes, Inserm, CIC 1414 (Centre d’Investigation Clinique de Rennes), Rennes, France; 8 Univ Rennes, CHU de Rennes, Inra, Inserm, Institut NUMECAN – UMR_A 1341, UMR_S 1241, Rennes, France; 9 Service d’Anesthésie-Réanimation, Hôpital Louis Pradel, Hospices Civils de Lyon, Bron, France; 10 EA 7426, Pathophysiology of Injury-Induced Immunosuppression, Hospices Civils de Lyon-Biomérieux-University, Claude Bernard Lyon 1, Lyon, France; 11 Division of Intensive Care, Geneva University Hospitals, the Faculty of Medicine, University of Geneva, Geneva, Switzerland; 12 AP-HP, Service de Médecine Intensive Réanimation, Hôpitaux Universitaires Henri-Mondor, Créteil, France; 13 Univ Paris Est Créteil, CARMAS, 94010 Créteil, France; 14 Service de Médecine Intensive–Réanimation, Hôpital Universitaire de Lille, Lille, France; 15 Service de Médecine Intensive - Réanimation, AP-HM, Hôpital Nord, Marseille, France; 16 Service de Réanimation Polyvalente, Centre Hospitalier Côte Basque et Service d’Anesthésie Réanimation Thoraco-Abdominale, CHU de Bordeaux, Université de Bordeaux, Bordeaux, France; 17 Department of Intensive Care, Hôpital Universitaire de Bruxelles (HUB), Brussels, Belgium; 18 Experimental Laboratory of Intensive Care, Université Libre de Bruxelles, Brussels, Belgium; 19 INSERM Umrs_1166-Ican, Institute of Cardiometabolism and Nutrition, Paris, France

**Keywords:** ECMO, Cannula-related infection, Hospital-acquired infection, Bloodstream infection

## Abstract

**Objective:**

Only few data regarding epidemiology and management of ECMO cannula-related infections (ECMO-CRIs) exist. The aim of our study was to describe their epidemiology and prognosis, and to evaluate factors associated with outcome.

**Methods:**

We performed a multicenter retrospective study in 12 European ICUs, including patients with ECMO-CRI, defined as a clinical suspicion plus a positive bacterial sample of ECMO-cannulation site. Primary objective was to describe ECMO-CRI characteristics and outcomes. Secondary objectives were to evaluate the rates of infection recurrence, their risk factors, and to evaluate the impact of antimicrobial treatment duration on outcome.

**Results:**

During the study period, 109 patients with ECMO-CRI (78 having concomitant positive blood culture with the same pathogen) were included. Pathogens responsible for infections were predominantly Enterobacteriaceae, coagulase-negative *Staphylococcus* and *Enterococcus* spp., and 42% of episodes were polymicrobial. Rates of infection recurrence was 13% and ICU-mortality rate was 51%. Risk factors for death were concomitant bloodstream infection with same pathogen and septic shock Patients with antibiotic course ≤ 8 days had similar infection recurrence rate and outcomes (including mortality) than patients with prolonged (> 8 days) antibiotic course.

**Conclusion:**

ECMO-CRIs are frequently associated with BSI and frequently polymicrobial. Duration of antimicrobial treatment for ECMO-CRI ≤ 8 days does not seem to be associated with an increased risk of recurrence or death, as compared to longer treatment.

**Supplementary Information:**

The online version contains supplementary material available at 10.1186/s13613-025-01446-y.

## Introduction

Extracorporeal membrane oxygenation (ECMO) is a complex therapy associated with potential life-threatening complications [1]. Infections are one of the main complications, occurring in 42–64% of ECMO patients [2–6]. Ventilator-associated pneumonia (VAP) is the most frequent one, but other ECMO-related infections (cannula-related infection (CRI), associated or not with bloodstream infection (BSI)) are frequent and may lead to increased morbidity/mortality. However, data regarding ECMO-CRI are scarce. In a retrospective large cohort of nosocomial infections in ECMO-patients, Schmidt et al. found that ECMO-CRI represents 10% of them [2], whereas Grasselli et al. found a 3% rate of CRI [5]. Discriminating cannula colonization from infection is sometimes challenging, since traditional clinical and biological signs of infection are frequently absent in ECMO patients.

Additionally, little is known about bacterial epidemiology of CRI, since published studies mostly focus on pediatric patients [7, 8]. In addition, no consensus on diagnostic criteria or management of ECMO-CRI exists, and little is known about their impact on prognosis. Data showed that long ECMO run (up to 10 days) were associated with increased rate of CRI, and that these infections were associated with prolonged hospital stay [8, 9]. Practices regarding treatment of these infections are heterogeneous [10], and optimal duration of antimicrobial treatment is unknown [11, 12].

We therefore design this study to describe the epidemiology, management and prognosis of ECMO-CRI, and to determine factors associated with outcomes, with a particular focus on the duration of antimicrobial treatment.

## Methods

### Centers

Thirteen high-volume ECMO centers (> 30 ECMO/year) were contacted and asked to participate. All were located in France, except 2 located outside France in Europe (Geneva and Brussels). Among the 13 centers contacted, 12 (10 in France and the 2 outside France) agreed to participate.

### Patients

Investigators from the 12 participating ICUs were invited to include their 10 most recent patients (starting from December 2022 and going back as far as January 2012) with ECMO-CRI (see definition below), that occurred either on veno-arterial- (VA) or veno-venous (VV)-ECMO. Since some centers had less than 10 CRI during the study period, other centers could include more than 10 patients.

### Study objectives

The primary objective was to describe the characteristics of ECMO-CRI, as defined hereafter, and its outcomes. Secondary objectives were to evaluate the rate of infection recurrence (see definition below), and to evaluate the impact of antimicrobial treatment duration on the outcome.

### Definitions

ECMO-CRI episodes were identified in each center by the investigator, based on patient’s chart. In all centers, criteria for ECMO-CRI were either inflammatory signs at cannula site or general signs of infection (fever, leukocytosis, hemodynamic impairment) combined with a positive microbial sampling of cannula site. Details regarding the centers ‘procedures for infection prevention and methods for ECMO-CRI diagnosis are given in the online supplement. ECMO-CRI was considered complicated by BSI when the same pathogen (same species, same resistance profile) was retrieved from blood culture and cannula sampling at the same time (within a 48-h time window). Patients with infection of the cannulation site occurring after ECMO removal were not included. Patients with cannula-colonization (positive bacterial sampling of cannula and without local signs or without BSI) were not included. Patients with primary BSI (BSI without portal of entry and without bacteriologically-proven ECMO-CRI) were not included in the present study. Although ECMO cannula may be the hidden portal of entry of such infection, we choose not to include them since we wanted to focus on CRI and its management.

Recurrence of ECMO-CRI was defined as a new infection episode (same definition as first episode) in a patient having developed a first episode, whatever the pathogen responsible for the second episode. Second episodes occurring after ECMO removal were also considered as recurrences. In these patients, recurrence was defined as a new infection of the cannula site (purulent drainage of the orifice or cellulitis) confirmed by a positive bacteriological sample. Patients without second episode of ECMO-CRI were considered as cured from infection.

Duration of antimicrobial treatment for the first episode was defined as the total duration of antimicrobial treatment effective against the pathogen(s) responsible for ECMO-related infection, including time on antibiotics given for another infection than ECMO-related infection. Antimicrobial treatment was considered appropriate when the incriminated microorganism was susceptible to the antibiotic administered on antimicrobial susceptibility testing. As an example, if a patient received amoxicillin for a ECMO-CRI due to wild-type *E. coli* had to be switched at day 7 of amoxicillin to a 7-day course of piperacillin-tazobactam for *Pseudomonas aeruginosa* VAP episode, he was considered to have received 14 days of effective antibiotic treatment for the initial ECMO-CRI.

Multi drug resistant organism (MDRO) was considered according to the definition of the European Society of Clinical Microbiology and Infectious Disease, as pathogens resistant to more than one antimicrobial agent [13].

We also recorded local cannula site complications after decannulation (hemorrhage, infection and thrombosis), mortality rate before ECMO weaning, duration of ECMO support and ventilatory support, length of ICU stays and ICU mortality rate. We then determined factors associated with recurrence.

### Statistical analysis

Categorical variables are expressed as percentages and continuous variables as median ant interquartile range (IQR). Chi-Square test and Fisher exact test were used to compare categorical variables and Mann–Whitney U test or Wilcoxon test for continuous variables.

To determine factors associated with ICU-mortality, uni- and multivariable analysis of factors associated with ICU death were performed using a Cox proportional model. Proportional assumption for Cox model was tested using scaled Schoenfeld residuals. Variables with a p-value < 0.2 in univariable analysis were entered into the model.

To further explore the impact of antimicrobial treatment duration of the first episode on outcome, we selected the 94 patients who survived at least 8 days after infection onset, and split them into 2 categories: short duration of antimicrobial treatment, i.e. ≤ 8 days, and long duration of treatment, i.e. > 8 days. To account for inter-group imbalances of baseline characteristics between patients with prolonged and short antibiotic duration, a propensity score overlap weighted analysis was performed [14]. Propensity score was calculated using a non-parsimonious model including the following variables: age, sex, comorbidities, reason for ECMO cannulation, BMI, COVID-19 status, antibiotic at ECMO cannulation, cardiac arrest at cannulation, type of ECMO, SOFA at ECMO cannulation and a modified SAPS II (in order to avoid redundancy with age, a modified SAPS II score was calculated with exclusion of the variable age). Propensity score ranked from 0 to 1 and corresponded for each patient to its probability to received prolonged antibiotic treatment. Then, in order to obtain 2 comparable groups and using an overlap weighted analysis, patients were attributed a weight of 1-PS (patients with prolonged antibiotic duration) or PS (patients with short treatment duration). Then the average effect of prolonged antibiotic duration was estimated by the difference between the overlap weighted populations. Analyses were performed with an alpha risk of 5%. Statistical analysis was performed with the statistical software R 4.1.1.

### Ethics

In accordance with current French law, informed written consent for demographic, physiologic and hospital-outcome data analyses was not obtained because this observational study did not modify existing diagnostic or therapeutic strategies. Nonetheless, patients and/or relatives were informed about the anonymous data collection and told that they could decline inclusion. The protocol was approved by the ethics committee of the Société de Réanimation de Langue Française on January, 15th, 2023 (registration no. CE-SRLF-22–074) for patients included in France and in Belgium, and the ethics committee of the Geneva University Hospitals for the patients included in Switzerland (registration n° 2023-00540). The database is registered by the Commission Nationale de l’Informatique et des Libertés (CNIL, registration no 2228484v 0).

## Results

Between January 2012 and December 2022, 109 ECMO patients with ECMO-CRI were included. Baseline patients’ characteristics are presented in Table 1. Patients were predominantly male (68%), young (median (IQR) age 52 (38–61 years), and 22 (20%) were immunosuppressed. We included higher number of patients with VA-ECMO (n = 77, 71%) than patients with VV-ECMO (n = 32, 29%).

**Table 1:** Baseline characteristics according to ICU

	Overall population n = 109	Alive n = 53	Death in ICU n = 56	p
Baseline characteristics				
Male sex	74 (67.9)	36 (67.9)	38 (67.9)	1.0
Age, years	52 [38–61]	50 [37–58]	55 [39–62]	0.29
BMI, kg/m^2^ *	28 [25–34]	28 [25–33]	28.5 [26–35]	0.69
Pre-existing conditions				
Chronic cardiac disease	39 (36)	18 (34)	21 (38)	0.85
Chronic renal disease	15 (14)	6 (11)	9 (16)	0.58
Chronic respiratory disease	15 (14)	4 (8)	11 (20)	0.12
Diabetes mellitus	34 (31)	17 (32)	17 (30)	1.0
Peripheral vascular disease	20 (18)	7 (13)	13 (23)	0.27
Immunocompromised †	22 (20)	9 (17)	13 (23)	0.57
Ongoing pregnancy	1 (1)	0	1 (2)	1.0
Admission SAPS2	41 [29–57]	42 [29–56]	41 [30–61]	0.75
Admission SOFA score	9 [4–12]	9 [6–11]	9 [4–12]	0.79
ECMO characteristics				
Veno-venous ECMO	32 (30)	12 (23)	20 (36)	0.19
Percutaneous cannulation	64 (66)	29 (67)	35 (65)	0.96
Ongoing antibiotics at ECMO start	44 (40)	17 (32)	27 (48)	0.13
SARS-Cov2 infection	22 (20)	8 (15)	14 (25)	0.29
Indication of ECMO				0.56
Cardiogenic shock	58 (53)	29 (55)	29 (52)	
ARDS	32 (29)	13 (25)	19 (34)	
Post-cardiotomy	11 (10)	5 (9)	6 (11)	
Septic shock	1 (1)	1 (2)	0 (0)	
E-CPR	5 (5)	4 (8)	1 (2)	
Other	2 (2)	1 (2)	1 (2)	
Cannulation by mobile unit	24 (23)	12 (24)	12 (22)	1.0

Data are expressed as median (IQR) or n (%)

BMI: Body Mass Index; SAPS2: Simplified acute physiology score 2; SOFA: Sepsis-related organ failure assessment; ECMO: Extra-corporeal membrane oxygenation; E-CPR: Extracorporeal cardiopulmonary resuscitation

^*^ Data available for 101/109 patients

^†^ Solid organ transplant recipients, active hematological malignancy or receiving immunosuppressant drug (including corticosteroids at a dose ≥ 0.5 mg/kg/d for ≥ 1 month)

### ECMO-CRI characteristics

ECMO-CRI characteristics (clinical presentation, pathogens responsible for infection and management of infection) are displayed in Table 2. Median [IQR] time between ECMO implantation and ECMO-CRI was 7 [5–13] days in patients having surgical cannula insertion, and 9 [6–15] days in patients with percutaneous cannula insertion (p = 0.08 for between-groups comparisons). Among the 109 episodes of ECMO-CRI, 78 (72%) had blood culture positive that yielded the same pathogen that the one retrieved at the cannula site. Although the majority (65%) had inflammatory signs at cannula insertion site, 41 had ECMO-CRI with concomitant BSI but without local sign of infection. Pathogens responsible for ECMO-CRI are shown on Table 2: *Enterobacteriaceae* were the most common pathogen responsible for these infections, followed by coagulase-negative *Staphylococcus* and *Enterococcus* spp.. Interestingly, 42 (39%) of episodes were polymicrobial. Regarding coagulase-negative *Staphylococcus,* 26 out of the 32 ECMO-CRI due to coagulase-negative *Staphylococcus* were associated with BSI with the same coagulase-negative *Staphylococcus*, and the 6 other were polymicrobial, coagulase-negative *Staphylococcus* being not the only pathogen responsible for ECMO-CRI.

**Table 2:** First ECMO-related infection episode characteristics

	Overall population n = 109	Alive n = 53	Death in ICU n = 56	p
Time between ECMO insertion and first episode, days	8 [6–15]	8 [5–13]	9 [6–19]	0.21
Concomitant BSI	78 (72)	31 (59)	47 (84)	0.006
Septic shock	61 (57)	17 (33)	44 (79)	< 0.001
Inflammatory sign at cannula insertion site	68 (65)	31 (61)	37 (69)	0.53
Pathogen				
*Staphylococcus aureus*	5 (5)	3 (6)	2 (4)	0.67
Methicillin resistant	0	0	0	-
*Coagulase-negative Staphylococcus*	32 (29)	13 (25)	19 (34)	0.3
*Streptococcus* spp	4 (4)	3 (6)	1 (2)	0.35
*Enterococcus* spp	33 (30)	16 (30)	17 (30)	1.0
Vancomycin resistant	0	0	0	-
*Enterobacteriaceae*	55 (51)	28 (53)	27 (48)	0.77
ESBL-producer	13 (12)	4 (8)	9 (16)	0.28
Carbapenem resistant	3 (3)	1 (2)	2 (4)	1.0
Non fermenting Gram-negative bacilli	20 (18)	11 (21)	9 (16)	0.7
Multiresistant NF-GNB	5 (5)	2 (4)	3 (5)	1.0
Anaerobe	5 (5)	2 (4)	3 (5)	1.0
MDRO	18 (17)	6 (11)	12 (21)	0.25
Polymicrobial	42 (39)	22 (42)	20 (36)	0.67
Time from diagnostic to empiric treatment, days	0 [0–0]	0 [0–0.5]	0 [0–0]	0.57
Appropriate empiric antimicrobial treatment	66 (69)	31 (62)	35 (76)	0.21
Time from infection onset to appropriate antimicrobial treatment, days	1 [0–2]	2 [1, 2]	0.50 [0–2]	0.01
Duration of antimicrobial treatment of first CRI, days *	10 [7–15]	11.50 [7–16]	10 [7–14]	0.12
Combination antimicrobial treatment	24 (22)	13 (25)	11 (20)	0.70
Oxygenator change during infection	27 (25)	13 (25)	14 (25)	1.0
Cannula site change for infection	22 (20)	10 (19)	12 (21)	0.93
Surgery needed for cannula site infection	32 (29)	18 (34)	14 (25)	0.41
ECMO-CRI recurrence	14 (13)	8 (15)	6 (11)	0.69
Time to recurrence, days †	6 [0–10]	7 [2–12]	0 [0–8]	0.09

Data are expressed as median [IQR] or n (%)

ECMO, Extra-corporeal membrane oxygenation. ICU, intensive care unit. CRI, Cannula related infection. ESBL, extended spectrum beta-lactamase. NF-GNB, Non fermenting Gram-negative bacilli. MDRO, multidrug resistant organism. BSI, bloodstream infection

^*^ Data available for 108/109 patients

^†^ Time from end of antimicrobial treatment of first episode to second episode onset

Details on treatment are reported in Table 2: median (IQR) duration of antimicrobial treatment was 10 (7–15) days, similar in patients with and without concomitant BSI (median (IQR) durations of 11 (7–15) and 10 (7–15) days, respectively, p = 0.4); 24 patients (22%) were treated using combination therapy during all the duration of antimicrobial treatment, 32 (29%) had surgery for their ECMO-CRI, and 22 (20%) had change of cannulation site for the infection.

### Outcomes

Among the 109 patients, 14 had a second episode of infection that occurred after a median (IQR) of 6 (0–10) days after the end of antimicrobial treatment of the first episode. Details regarding the outcomes and pathogens responsible for the second episode of infection are reported in Table 3. Among these recurrences, 7 (50%) occurred after ECMO removal, with a median [IQR] time from ECMO removal to recurrence of 5 [1–8] days.

**Table 3:** Outcomes

	Overall population n = 109
Recurrence of infection	14 (13)
Time to new infection, days^*^	6 [0–10]
Pathogen responsible for second episode	
*Staphylococcus aureus*	0
Coagulase-negative *Staphylococcus*	3 (21)
*Streptococcus* spp	1 (7)
*Enterococcus* spp.	6 (43)
Enterobacteriaceae	5 (36)
Non fermenting Gram-negative bacilli	2 (14)
MDRO	7 (50)
Polymicrobial	9 (64)
Complications on cannula site after decannulation	
Arterial thrombosis	6 (6)
Hemorrhage	4 (4)
Duration of ECMO support, days	15 [9–23]
Duration of invasive mechanical ventilation, days	20 [10–34]
ICU length of stay, days	26 [18–41]
Death on ECMO	33 (30)
ICU mortality rate	56 (51)

Data are expressed as median (IQR) or n (%)

ECMO, Extracorporeal membrane oxygenation. ICU, intensive care unit. MDRO, multidrug resistant organism

^*^ Time from end of antimicrobial treatment of first episode to second episode onset

Other outcomes are reported in Table 3. ICU-mortality rate was 51%. Uni- and multivariable analyses of factors associated with mortality are shown in Table 4: concomitant BSI and septic shock complicating ECMO-CRI were associated with ICU-mortality.

**Table 4:** Proportional Cox uni and multivariable analysis of factors for death in the 109 patients with cannula-related infection

	Univariable analysis			Multivariable analysis		
	HR	95% CI	p	HR	95% CI	p
Variables						
Age. per year	1.01	0.99–1.03	0.19	1.00	0.98–1.02	0.27
Male sex	0.98	0.56–1.72	0.95			
Body mass index. per point	1.01	0.97–1.04	0.79			
Indication for ECMO						
Cardiogenic shock						
Acute respiratory distress syndrome	1.06	0.59–1.89	0.85			
Post-cardiotomy	1.46	0.6–3.51	0.40			
Septic shock	0	0-∞	0.99			
E-CPR	0.3	0.04–2.23	0.24			
Other	0.72	0.1–5.28	0.75			
SARS-CoV2 infection	1.16	0.63–2.13	0.62			
Pre-existing conditions						
Chronic cardiac disease	1.21	0.7–2.07	0.49			
Chronic renal disease	1.44	0.71–2.94	0.32			
Chronic respiratory disease	2.01	1.04–3.89	0.03	1.49	0.74–3.00	0.51
Diabetes mellitus	0.95	0.54–1.68	0.86			
Peripheral vascular disease	1.54	0.83–2.88	0.17	1.27	0.62–2.60	0.94
Immunocompromised*	1.33	0.71–2.47	0.37			
Pregnancy	2.4	0.33–17.51	0.39			
Admission SAPS II score	1.00	0.99–1.02	0.68			
Admission SOFA score	1.01	0.95–1.07	0.86			
Veno-venous ECMO†	1.21	0.7–2.1	0.49			
Cardiac arrest during cannulation	0.66	0.28–1.55	0.34			
Site of cannulation						
Femoro-jugular						
Femoro-femoral	0.80	0.46–1.39	0.43			
Other	1.76	0.41–7.52	0.45			
Implantation by mobile unit	0.99	0.52–1.89	0.99			
Percutaneous cannulation	0.95	0.54–1.66	0.85			
Ongoing antibiotics at ECMO start	1.46	0.86–2.47	0.16	0.98	0.54–1.76	0.94
Associated bloodstream infection‡	2.7	1.32–5.51	0.006	2.33	1.09–5.00	0.03
Time from ECMO start to infection onset, per day	1.03	1–1.05	0.04	1.03	0.99–1.06	0.052
Inflammatory sign at cannula insertion site	1.08	0.61–1.92	0.79			
Septic shock	4.78	2.51–9.1	< 0.001	4.10	2.07–8.10	< 0.001
Time from diagnostic to empiric treatment. per day	0.89	0.70–1.13	0.35			
Time from infection onset to appropriate antimicrobial treatment. per day	0.83	0.69–1.00	0.05	0.95	0.78–1.15	0.58
Combination therapy	0.78	0.4–1.51	0.46			

BMI. body mass index. ECMO. extracorporeal membrane oxygenation. SAPS 2. Simplified acute physiology score 2. SOFA. Sepsis-related organ failure assessment. E-CPR. Extracorporeal cardiopulmonary resuscitation. SARS CoV-2: severe acute respiratory syndrome coronavirus-2. MDRO: multi-drug resistant organism

^*^ Solid organ transplant recipients. active hematological malignancy or receiving immunosuppressant drug (including corticosteroids at a dose ≥ 0.5 mg/kg/d for ≥ 1 month)

^†^ As compared to veno-arterial ECMO

^‡^ As compared to cannula-related infection without associated bloodstream infection

### Impact of antimicrobial treatment duration

To explore the impact of antimicrobial treatment duration of the first episode on outcome, we selected the 94 patients who survived at least 8 days after infection onset, and analyze them according to the duration of antimicrobial treatment, i.e. ≤ 8 days vs. > 8 days. Results of this analysis are presented in the online supplement: 31 patients had a short (≤ 8 days) duration of antimicrobial treatment and 63 had a long (> 8 days) duration of antimicrobial treatment; with median (IQR) durations of treatment of 7 (6–8) and 15 (11–18) days, respectively. Characteristics of patients were similar between patients with short or long duration of treatment (see eTable 1, online supplement). Data regarding infection characteristics, microbiological data and management of infection according to duration of antimicrobial treatment are presented in eTable 2 (online supplement). Patients with long duration of treatment had more frequently surgery on cannulation site than patients with short duration; however the outcomes were similar between the 2 groups; particularly the recurrence rates and ICU mortality rates were similar.

Then, as detailed in the method section, a propensity score was constructed to reduce imbalance between groups, in order to evaluate through an overlap weighting analysis, the effect of prolonged (> 8) versus standard (≤ 8 days) duration of antimicrobial treatment. Weighted groups were well balanced and standardized mean differences were < 0.1 for all variables (see eFigure 1, online supplement). Considering a cox proportional hazard model with propensity score overlap weighting, patients treated for more than 8 days exhibited significantly higher hazard of ICU mortality compared to those treated for 8 days or less, with an HR of 1.72 (95% CI, 1.02–2.90; p = 0.043).

## Discussion

In this multicenter study of 109 patients with ECMO-CRI, we found that more than 2/3 of patients with cannula-related infection had associated BSI due to the same pathogen. Main pathogens responsible for ECMO-CRI were *Enterobacteriaceae*, coagulase-negative *Staphylococcus* and *Enterococcus* spp., and 39% of these infections involved more than one pathogen. Outcomes of ECMO-CRI were globally poor, since 13% experienced infection recurrence and 51% died in the ICU. Factors associated with death were associated bloodstream infection and ECMO-CRI complicated by septic shock. Interestingly, patients with a short duration of antimicrobial treatment (≤ 8 days) seem to have better outcomes than patients with long duration of antimicrobial treatment (> 8 days). Unfortunately, the small number of recurrences (n = 14) did not allow us to explore factors associated with recurrence.

This is the largest multicenter cohort regarding ECMO-CRI in adults, and the first that describes its epidemiology and outcomes. Among the previous observational studies evaluating infection in ECMO patients, small case series showed that *Enterobacteriaceae* and non-fermenting Gram negative bacilli were the most frequent pathogen involved in ECMO-associated infection [8, 11, 15], while other highlighted the central role of *Enterococcus* spp. in nosocomial infections [2, 5]. However, most infections recorded in these studies were not specifically ECMO-related, i.e., included VAP or urinary tract infection [2, 5]. Among them, only few ECMO-CRIs were documented [2, 5, 15, 16]. Nevertheless, our findings are in agreement with these results, since the main pathogens responsible for ECMO-CRI were *Enterococcus* spp. and *Enterobacteriaceae.* Of note, coagulase-negative *Staphylococcus* represented 30% of CRI in our study, whereas a previous study that included 21 ECMO-CRI episodes reported a 19% rate of coagulase-negative *Staphylococcus* [2]. Last, pathogens responsible for infections in our study were less frequently MDRO than in others, [5], probably related to the relatively low rate of MDRO in French ICUs, as compared to other European countries.

Few data regarding outcomes of patients with ECMO-CRI are available. A small single center cohort study showed that ECMO patients who developed infection had significantly higher mortality rate than patients without infection (40.4% vs. 20.0%, respectively, p = 0.037). However this study reported only 3 ECMO-CRIs and 5 BSIs [5]. Moreover, these results were not confirmed in others [2, 11, 15]; Schmidt et al., in their cohort study of 220 ECMO patients, found that infected and non-infected patients had similar mortality rate (51% vs. 62%, respectively, p = 0.15). Although our study was not designed to compare patients with and without CRI, ICU-mortality rate of our patients was similar to that observed in the Schmidt study [2]. One of the originalities of our work was to focus on other outcomes, and in particular infection recurrence. Although we were unable to explore factors associated with infection recurrence, due to the small number of patients with recurrence, we show here that recurrences were also frequently polymicrobial,

Management of ECMO-CRI may be challenging, with specific issues regarding antimicrobial treatment duration and need for cannula change to cure infection. To date, no data focusing on these issues exist in the literature. We showed here that neither antimicrobial treatment duration, nor combination therapy, nor cannula change were associated with an increased risk of mortality. Specifically, patients with short duration of antimicrobial treatment (≤ 8 days) seem to have better outcome than patients with prolonged (> 8 days) duration of antimicrobial treatment. This observation is in line with recent data on infections in ICU patients, which showed that short durations of antimicrobial treatment, even for severe infections, are not associated with worse outcomes, but may even be associated with better outcomes [17].

Our study has several limitations that should be underlined. Firstly, selection of patients with ECMO-CRI was performed retrospectively, based on patient’s chart, therefore some ECMO-CRI episodes could have been missed, and we are unable to calculate the incidence of ECMO-CRI. However, this was not the purpose of our study. Secondly, due to its retrospective design, we cannot be sure that all parameters recorded in the present study were reported in the patient chart, including clinical data regarding the ECMO-CRI episode (inflammatory signs at ECMO cannulation site…). Moreover, the retrospective design limits the interpretation of outcomes. Therefore, our results should be interpreted cautiously. Thirdly, there is no clear definition of CRI and no consensus on the diagnosis process (which sample, which type of sampling…). We used criteria described in previous papers, but these criteria may vary from one study to another [2, 18–20], and were different among centers having included patients. Additionally, center effect was not taken into account in our study. However, since all included patients had either ECMO-CRI concomitant BSI or local signs of infection, our population is homogenous, without patients with ECMO-cannula colonization. On the other side, patients developing BSI without local signs (at the cannula insertion site) may have ECMO-CRI. Since we excluded patients who were not sampled, we could have missed some patients with ECMO-CRI. Fourthly, we excluded patients with primary BSI and therefore could have miss some patients with ECMO-CRI: indeed, some patients with primary BSI could have in fact ECMO-CRI (not diagnosed for several reasons). However, we preferred to include only patients with bacteriologically proven ECMO-CRI. Fifthly, we had a large proportion of patients with CRI due to coagulase-negative *Staphylococcus*, that could be considered as colonization rather than infection. However, 26 out of the 32 ECMO-CRI due to coagulase-negative Staphylococci were associated with BSI with the same coagulase-negative *Staphylococcus*, and the 6 other were polymicrobial, coagulase-negative *Staphylococcus* being not the only pathogen responsible for ECMO-CRI. Therefore, we believe that all episodes reported here due to coagulase-negative Staphylococci were actually due to this pathogen (alone or in combination with others). Sixthly, the relationship between duration of antimicrobial treatment and outcome should be interpreted with caution: indeed, treatment duration might depend on factors as the time on ECMO after treatment initiation, possibly on the responsible pathogens, association of soft tissue infection, time to surgery, and other cofounding factors. Finally, even if this is a multicenter cohort, our study mostly performed in French ICUs. We cannot be sure that similar results would be observed in ICUs with different bacterial ecologies.

## Conclusion

ECMO-CRI is frequently polymicrobial, mainly due to Enterobacteriaceae, coagulase-negative *Staphylococci* and *Enterococcus* spp, and frequently associated with BSI. ECMO-CRI management, and more specifically duration of antimicrobial treatment does not seem to influence mortality.

## Supplementary Information

Additional file 1

## Data Availability

The datasets generated during the current study are available from the corresponding author on reasonable request.

## Abbreviations

BSI: Bloodstream infection
CRI: Cannula-related infection
ECMO: Extracorporeal membrane oxygenation
ICU: Intensive care unit
IQR: Interquartile range
VAP: Ventilated-associated pneumonia
VA: Veno-arterial
VV: Veno-venous
