# Supplementary material for: Characteristics and outcomes of ECMO cannula-related infections: a European multicenter retrospective study
Source: Ann Intensive Care. 2025 Mar 20;15:36. doi: 10.1186/s13613-025-01446-y (PMC11926307; doi:10.1186/s13613-025-01446-y)
Supplement: Supplementary file 1 — Additional file 1 [file 13613_2025_1446_MOESM1_ESM.docx]

**Characteristics and outcomes of ECMO-related infections:**

**an European multicenter retrospective study**

S Ortuno, N Massart, C Vidal, E de Montmollin, A Bouglé, N Nesseler, F Bidar, B Assouline, P Masi, S Henri, S Hraiech, H Rozé, F Manicone, CE Luyt

**Online supplement**

**Methods**

*Prevention of CRI infection*

Six centers used chlorhexidine-impregnated dressing on cannula site. Except in case of active bleeding, dressing were changed every 24 hrs. in one center, every 48 hrs. in 2 centers, every 96 hrs. in one center and every 7 days in all other centers. Four centers performed systematic surveillance blood cultures.

*CRI diagnosis*

In all centers, CRI was defined as the combination of the 2 following parameters:

- Local (cannula-site) inflammatory signs and/or general signs suggestive of infection (fever, leukocytosis, hemodynamic impairment)
- Pathogen(s) retrieved from a cannula-site sample

Types of cannula-site sampling varied across centers: 9 centers performed a deep-site sampling, using a needle inserted along the cannula for 7 or during surgical revision for one; and the remaining 3 centers performed a superficial sampling using a swab.

**eTable 1.** Baseline characteristics of the 94 patients alive at day 8 after infection onset. according to the duration of effective antimicrobial treatment (short. ≤8 days or long. >8days)

|  | Short (≤8 days) duration  N = 31 | Long (>8 days) duration  N = 63 | p |  |
| --- | --- | --- | --- | --- |
| Baseline characteristics |  |  |  |  |
| Male sex | 50 [32-57.50] | 52 [40.50-61.50] | 0.19 |  |
| Age, years | 21 (67.7) | 44 (69.8) | 1.0 |  |
| BMI, kg/m² ^a^ | 32 [26-39] | 28 [24-33] | 0.12 |  |
| Pre-existing conditions |  |  |  |  |
| Chronic cardiac disease | 5 (16.1) | 26 (41.3) | 0.03 |  |
| Chronic renal disease | 4 (12.9) | 8 (12.7) | 1.0 |  |
| Chronic respiratory disease | 4 (12.9) | 6 (9.5) | 0.89 |  |
| Diabetes mellitus | 10 (32.3) | 19 (30.2) | 1.0 |  |
| Peripheral vascular disease | 3 (9.7) | 13 (20.6) | 0.30 |  |
| Immunocompromised^b^ | 5 (16.1) | 13 (20.6) | 0.81 |  |
| Ongoing pregnancy | 0 (0.0) | 1 (1.6) | 1.0 |  |
| Admission SAPS2 | 43 [31-57] | 41.50 [28-62] | 0.79 |  |
| Admission SOFA score | 8 [4.50-12] | 9 [4-11] | 0.85 |  |
| ECMO characteristics |  |  |  |  |
| Veno-venous ECMO | 14 (45.2) | 16 (25.4) | 0.09 |  |
| Percutaneous cannulation | 21 (75.0) | 34 (61.8) | 0.34 |  |
| Ongoing antibiotics at ECMO start | 13 (41.9) | 26 (41.3) | 1.0 |  |
| SARS-Cov2 infection | 10 (32.3) | 11 (17.5) | 0.18 |  |
| Indication of ECMO |  |  | 0.2 |  |
| Cardiogenic shock | 13 (41.9) | 36 (57.1) |  |  |
| ARDS | 14 (45.2) | 16 (25.4) |  |  |
| Post-cardiotomy | 1 (3.2) | 6 (9.5) |  |  |
| Septic shock | 1 (3.2) | 0 (0.0) |  |  |
| E-CPR | 1 (3.2) | 4 (6.3) |  |  |
| Other | 1 (3.2) | 1 (1.6) |  |  |
| Cannulation by mobile unit | 7 (24.1) | 13 (21.0) | 0.95 |  |

Results are expressed as n (%) or median (IQR)

Abbreviations: BMI. body mass index. ECMO. extracorporeal membrane oxygenation. SAPS 2. Simplified acute physiology score 2. SOFA. Sepsis-related organ failure assessment. E-CPR. Extracorporeal cardiopulmonary resuscitation.

* Solid organ transplant recipients. active hematological malignancy or receiving immunosuppressant drug (including corticosteroids at a dose≥0.5 mg/kg/d for≥1 month)

**eTable 2**. Characteristics, microbiology and management of cannula-related infection in the patients alive at day 8 after infection onset, according to the duration of effective antimicrobial treatment (≤8 days or >8days)

|  | Short (≤8 days) duration  N = 31 | Long (>8 days) duration  N = 63 | p |
| --- | --- | --- | --- |
| Time between ICU admission and first episode, days | 10 [6-14] | 8 [5-15] | 0.504 |
| Concomitant BSI | 21 (67.7) | 43 (68.3) | 1.000 |
| Septic shock | 12 (41.4) | 35 (55.6) | 0.299 |
| Inflammatory sign at cannula insertion site | 19 (65.5) | 42 (67.7) | 1.000 |
| Pathogen |  |  |  |
| Staphylococcus aureus | 0 (0.0) | 3 (4.8) | 0.548 |
| Methicillin resistant | 0 | 0 |  |
| Coagulase-negative Staphylococcus | 7 (22.6) | 19 (30.2) | 0.475 |
| Streptococcus spp | 2 (6.5) | 2 (3.2) | 0.596 |
| Enterococcus spp | 11 (35.5) | 18 (28.6) | 0.657 |
| Vancomycin resistant | 0 | 0 |  |
| Enterobacteriaceae | 17 (54.8) | 30 (47.6) | 0.661 |
| ESBL-producer | 3 (9.7) | 8 (12.7) |  |
| Carbapenem resistant | 1 (3.2) | 2 (3.2) |  |
| Non fermenting Gram-negative bacilli | 5 (16.1) | 14 (22.2) | 0.676 |
| Multi drug resistant | 2 (6.5) | 3 (4.8) | 1.0 |
| Anaerobe | 1 (3.2) | 3 (4.8) | 1.000 |
| MDRO | 5 (16.1) | 11 (17.5) | 1.000 |
| Polymicrobial | 12 (38.7) | 25 (39.7) | 1.000 |
| Time from diagnostic to empiric treatment, days | 0 [0-1] | 0 [0-0] | 0.297 |
| Appropriate empiric antimicrobial treatment | 18 (66.7) | 36 (66.7) | 1.000 |
| Time from infection onset to appropriate antimicrobial treatment, days | 2 [0-3] | 2 [0-2] | 0.423 |
| Duration of antimicrobial treatment of first ECMO-related infection, days | 7 [6-8] | 15 [11-18] | <0.001 |
| Combination antimicrobial treatment | 7 (22.6) | 15 (23.8) | 1.000 |
| Oxygenator change during infection | 5 (16.1) | 18 (28.6) | 0.287 |
| Cannula site change for infection | 6 (19.4) | 13 (20.6) | 1.000 |
| Surgery needed for cannula site infection | 5 (16.1) | 24 (38.1) | 0.054 |
| Recurrence of infection | 3 (9.7) | 11 (17.5) | 0.491 |
| Time to new infection, daysa | 0 [0-8.25] | 6 [0-10] | 0.493 |
| Pathogen responsible for second episode |  |  |  |
| Staphylococcus aureus | 0 | 0 |  |
| Coagulase-negative Staphylococcus | 1 (33.3) | 2 (18.2) | 1.000 |
| Streptococcus spp | 1 (33.3) | 0 (0.0) | 0.470 |
| Enterococcus spp. | 1 (33.3) | 5 (45.5) | 1.000 |
| Enterobacteriaceae | 1 (33.3) | 4 (36.4) | 1.000 |
| Non fermenting Gram-negative bacilli | 0 (0.0) | 2 (18.2) | 1.000 |
| MDRO | 1 (33.3) | 6 (54.5) | 1.000 |
| Polymicrobial | 2 (66.7) | 7 (63.6) | 1.000 |
| Complications on cannula site after decannulation |  |  |  |
| Cannula site infection | 2 (6.5) | 14 (22.2) | 0.105 |
| Arterial thrombosis | 2 (6.5) | 4 (6.3) | 1.000 |
| Hemorrhage | 1 (3.2) | 2 (3.2) | 1.000 |
| Duration of ECMO support, days | 15 [9-29] | 15 [9-20] | 0.533 |
| Duration of invasive mechanical ventilation, days | 20 [14-46] | 20 [7-30] | 0.173 |
| ICU length of stay, days | 30 [21-58] | 28 [20-41] | 0.391 |
| Death on ECMO | 7 (22.6) | 17 (27.0) | 0.835 |
| ICU mortality rate | 10 (32.3) | 32 (50.8) | 0.139 |

Results are expressed as n (%) or median (IQR)

Abbreviations: ECMO. extracorporeal membrane oxygenation. ESBL. extended-spectrum betalactamase. ICU. intensive care unit.

eFigure1. Standardized mean difference of baseline characteristics before and after overlap weighting analysis
